# A Coupled EXAFS–Molecular Dynamics Study on PuO_2_^+^ and NpO_2_^+^ Hydration: The Importance of Electron Correlation in Force-Field Building

**DOI:** 10.1021/acs.inorgchem.2c00461

**Published:** 2022-05-26

**Authors:** Gema Raposo-Hernández, José M. Martínez, Rafael R. Pappalardo, Christophe Den Auwer, Enrique Sánchez Marcos

**Affiliations:** † Department of Physical Chemistry, University of Seville, 41012 Seville, Spain; ‡ Université Côte d’ Azur, CNRS, ICN, 06108 Nice, France

## Abstract

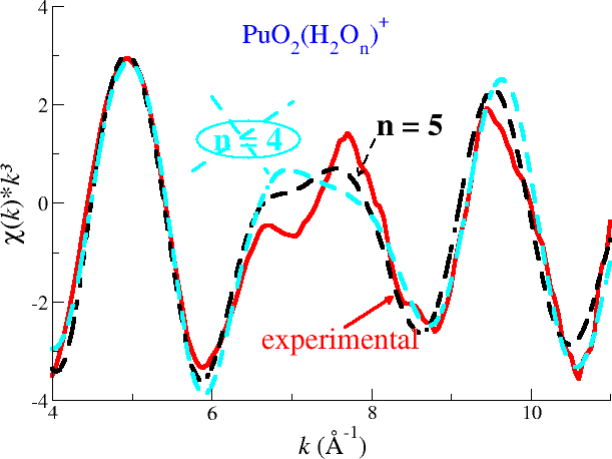

The physicochemical properties of the monovalent actinyl cations, PuO_2_^+^ and NpO_2_^+^, in water have been studied by means of classical molecular dynamic simulations. A specific set of cation-water intermolecular potentials based on ab initio potential energy surfaces has been built on the basis of the hydrated ion concept. The TIP4P water model was adopted. Given the paramagnetic character of these actinyls, the cation–water interaction energies were computed from highly correlated wave functions using the NEVPT2 method. It is shown that the multideterminantal character of the wave function has a relevant effect on the main distances of the hydrated molecular cations. Several structural, dynamical, and energetic properties of the aqueous solutions have been obtained and analyzed. Structural RDF analysis gives An–O_yl_ distances of 1.82 and 1.84 Å and An–O(water) distances of 2.51 and 2.53 Å for PuO_2_^+^ and NpO_2_^+^ in water, respectively. Experimental EXAFS spectra from dilute aqueous solutions of PuO_2_^+^ and NpO_2_^+^ are revisited and analyzed, assuming tetra- and pentahydration of the actinyl cations. Simulated EXAFS spectra have been computed from the snapshots of the MD simulations. Good agreement with the experimental information available is found. The global analysis leads us to conclude that both PuO_2_^+^ and NpO_2_^+^ cations in water are stable pentahydrated aqua ions.

## Abstract 2

Force fields to describe PuO_2_^+^ and NpO_2_^+^ in water based on NEVPT2 potential energy surfaces have been built and applied via classical MD simulations to predict physicochemical properties in solution such as hydration enthalpy, diffusion coefficient, or EXAFS spectrum. The reliability of the methodology has been supported by the good comparison of the scarce experimental information with the theoretically derived values.

## Introduction

The actinyl forms of actinoids are their trans-dioxo molecular cations, AnO_2_^+/2+^, present in the high oxidation states, V and VI.^1^ They exhibit high solubilities in water what leads to a rich solution chemistry in a wide set of scenarios where their chemical behavior may be quite different: ligand complexation, hydrolysis processes coupled to acidity or basicity of the medium, adsorption on surfaces, polymerization, and others.^2−7^ It is of primary interest the proper structural characterization of the hydration of such actinyls in order to further understand their physicochemical properties in the different environments that they may face. It has been shown that these actinyls in water are surrounded by water molecules defining stable aqua ions, [AnO_2_(H_2_O)_*n*_]^+/2+^, being generally accepted to have a hydration number of five,^3,8−13^ although four has also been proposed in some cases.^14−16^ The actinyl aqua ion chemistry is intimately joined to nuclear technology due to its role in spent nuclear fuel, reprocessing, temporary and permanent storage, and environmental speciation.^6,17^

The structural characterization of these cations has been conducted by both experimental and theoretical techniques. Among the experimental ones, the technique giving a more direct information is the X-ray absorption spectroscopy (XAS), in particular the extended X-ray absorption fine structure (EXAFS).^4,17−20^ The great advantage of this technique is its ability to supply short-range structural information around an specific atom, the absorbing atom, with a structural precision of 0.01 Å for the first coordination shell distance and one unit in the coordination number.^10,12,19,21,22^ Given that no long-range order is needed and submillimolar concentrations of the absorbing atom can be detected, EXAFS is a really powerful and very suitable tool for the study of solutions containing actinoid cations.^4,17,20,23^

Among the theoretical techniques, quantum-mechanical methods and computer simulations have also provided valuable information. In the first case, the studies usually combine the molecular cation with a small number of water molecules forming the hydrated ion and adding solvent boundary conditions by means of a continuum solvation model.^24−29^ A general solvent molecular description can be reached by using statistical techniques, either assuming ab initio molecular dynamics (AIMD) at reliable QM levels or classical MD simulations employing in this case reliable force fields.

The combination of XAS spectroscopy and MD simulations has been revealed as an useful strategy^30−33^ to refine the structural properties of solutions when the standard fitting of the experimental spectra are clouded by different factors as complexity of the system, low concentrations, spectroscopical phenomena as multiexcitations, low signal/noise ratio, and others.^34^ The good reproduction of an experimental spectrum by means of the use of the structural information derived from a statistical simulation has a double consequence.^23^ On the one hand, it allows access to a direct EXAFS structure assignment provided by the atomistic picture of the statistical trajectory. On the other hand, the agreement shows the ability of the interaction potentials employed in the statistical simulation when using classical force fields or the quantum-mechanical level in AIMD simulations to describe properly the system.

Within the actinyl series, PuO_2_^+^ has been scarcely studied due to its trend to disproportionate to lower, e.g., Pu^4+^, and higher, e.g., PuO_2_^2+^, oxidation states. EXAFS studies have dealt with PuO_2_^+^ acidic aqueous solutions.^14,15,20^ The main distances, Pu–O_yl_ and Pu–O_I_, and the hydration number are collected in Table 1. The EXAFS fitting supplies a reduced range for the main distances, Pu–O_yl_ and Pu–O_I_, but the coordination numbers proposed vary from 3.3 to 5.3. Bearing in mind the typical uncertainty in the coordination number determination from EXAFS fitting due to multiparameter correlation, additional information must be included to elucidate this issue. Some QM calculations had concluded that coordination number is 5 with distances quite different from those obtained by EXAFS, as collected in Table 1.^35^ Dalodière et al.,^20^ in a recent study on the PuO_2_^+^ aqua ion, showed an interesting synthesis method of this species based on sonochemistry which allowed them to reach millimolar PuO_2_^+^ solutions free of other Pu oxidation states. They recorded the EXAFS spectrum of this species and compared it with simulated spectra computed from the QM [PuO_2_(H_2_O)_5_]^+^(H_2_O)_10_ and [PuO_2_(H_2_O)_4_]^+^(H_2_O)_8_ clusters, obtained by B3LYP optimizations and Debye–Waller factors computed from the QM frequencies of these clusters. They concluded that the best theoretical–experimental agreement corresponds to the PuO_2_^+^ tetrahydrate.

**Table 1:** Gas-Phase QM and Force-Field Optimizations, MD First-Shell Distances at 300 K, and Debye–Waller factors (σ^2^)^a^

method	CN	*R*_An–Oyl_ (Å)	σ_An–Oyl_^2^ (Å^2^)	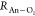 (Å)	σ^2^ (An−O_I_)	ref
[PuO_2_(H_2_O)*_n_*]^+^						
QM(NEVPT2)	5	1.806		2.506		this work
QM(NEVPT2)	4	1.800		2.448		this work
POT5(NEVPT2)	5	1.805		2.505		this work
POT5(NEVPT2)	4	1.799		2.437		this work
POT4(NEVPT2)	4	1.795		2.450		this work
MD_POT5 (300 K)	5	1.822	0.0007	2.507	0.008	this work
MD_POT4 (300 K)	4	1.809	0.00035	2.454	0.0013	this work
EXAFS	4	1.821	0.030	2.48	0.072	Panak et al.^14^
EXAFS	3.3	1.81	0.0020	2.47	0.0044	Di Giandomenico et al.^15^
EXAFS	5.3	1.81	0.002	2.47	0.009	Dalodière et al.^20^
QM(B3LYP)	5	1.808		2.61		Hay et al.^25^
QM(PBE)	5	1.86		2.53		Rizhkov et al.^35^
QM (B3LYP)	5	1.76		2.53		Pomogaev et al.^58^
MD_POT5 (300 K)	5	1.77		2.56		Pomogaev et al.^58^
MD/PBE (300 K)	5	1.82		2.55		Odoh et al.^16^
MD/PBE (300 K)	4	1.85		2.47		Odoh et al.^16^
[NpO_2_(H_2_O)*_n_*]^+^						
QM(NEVPT2)	5	1.831		2.522		this work
QM(NEVPT2)	4	1.825		2.466		this work
POT5(NEVPT2)	5	1.825 (1.83)		2.529 (2.52)		this work (Pérez-Conesa et al.)^38^
MD_POT5 (300 K)	5	1.842 (1.84)	0.0007 (0.0007)	2.528 (2.54)	0.008 (0.011)	this work (Pérez-Conesa et al.)^38^
EXAFS	5	1.822	0.002	2.488	0.006	Reich et al.^9^
EXAFS	5.2	1.84	0.002	2.49	0.007	Ikeda et al.^12^
EXAFS	4.4	1.83	0.0056	2.51	0.0040	Di Giandomenico et al.^15^
HEXS	5	1.82		2.46		Skanthakumar et al.^13^
QM(B3LYP)	5	1.81		2.61		Hay et al.^25^
QM(MP2)	5	1.81		2.52		Tsushima et al.^24^
QM (B3LYP)	5	1.79		2.55		Pomogaev et al.^58^
QM (B3LYP)	5	1.79		2.59		Danilo et al.^28^
QM (B3LYP)	5	1.78		2.59		Pérez-Conesa et al.^38^
MD (300 K)	5	1.80		2.54		Pomogaev et al.^58^

a All experimental EXAFS are obtained at highly acidic pH, with non-coordinating counterions.

In previous works,^36−38^ we have conducted classical MD simulations of actinyls, AnO_2_^+/2+^, in water using interaction potentials based on first-principles QM calculations. EXAFS and XANES spectra have been simulated using the structural information and the theoretical scattering phases and amplitude functions computed by the ab initio FEFF code (v.9.6).^39^ Whereas the theoretical–experimental agreement was quite satisfactory for UO_2_^2+^, NpO_2_^2+^, and PuO_2_^2+^ using force fields derived from B3LYP potential energy surfaces, the NpO_2_^+^ case was not satisfactory.^38^ This fact compelled us to develop for NpO_2_^+^ a new force field based on QM wave functions with explicit inclusion of the dynamic and nondynamic electron correlation, as NEVPT2 method does.^40,41^ The simulated EXAFS spectrum gave a fair comparison with the experimental spectra. As far we know, there is not a simulated spectra based on statistical computations for PuO_2_^+^, with the coordination number and geometrical parameter remaining controversial.

The aim of this work is to confirm the importance of the use of multideterminantal wave functions as reference QM computations to provide accurate enough force fields when dealing with a higher multiplet open-shell system, as it is that of the PuO_2_^+^ cation. Likewise, we envisage to develop and test a simpler formulation of the actinyl potential model. For these reasons, we have built a new NpO_2_^+^–H_2_O interaction potential to double check the validity of this new formulation. A revisited analysis of former experimental EXAFS spectra of PuO_2_^+^ and NpO_2_^+^ aqueous solutions^15^ has also been carried out on the light of the theoretical results.

## Methods

### Quantum Chemical Calculations

**Figure 1:**
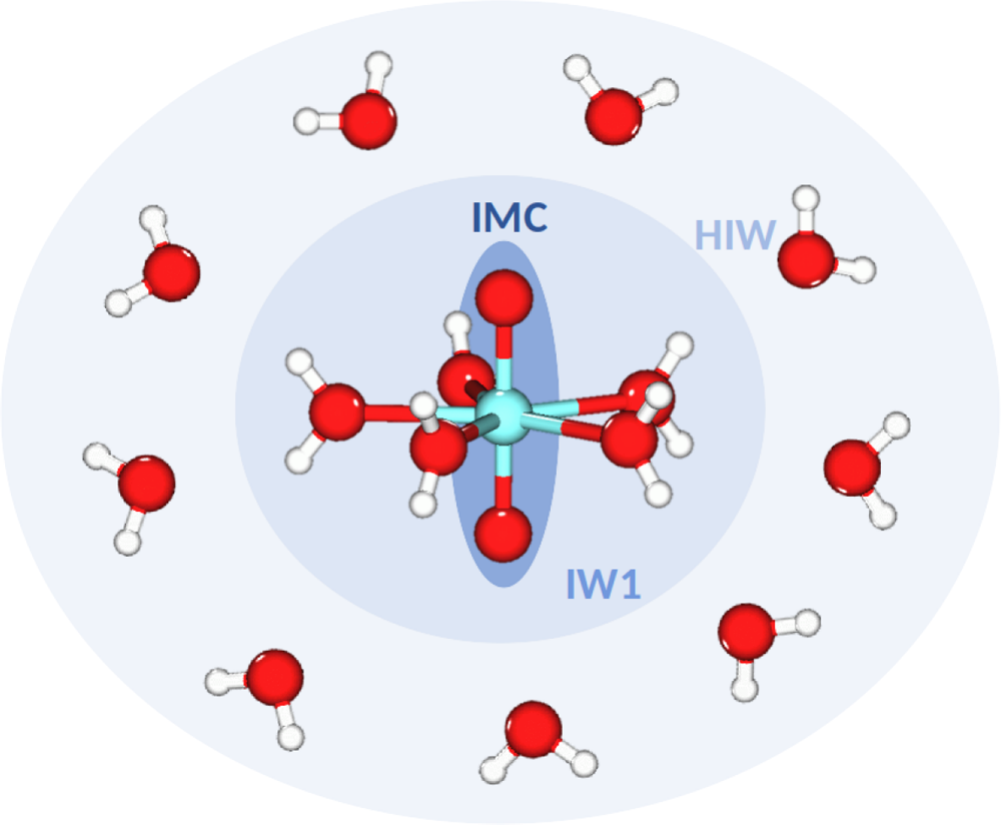
Interaction potentials defined in the system: IMC (intramolecular cation interaction), IW1 (molecular cation–water first-shell interactions, and HIW (hydrated ion–bulk water interactions). The water–water interactions are described by the TIP4P model.^52^

A main motivation of this study is to get insight into the impact that static and dynamic electron correlation may have on the structure and the dynamical and structural disorder of the close environment of the actinyls. Multireference NEVPT2^40−42^ calculations, which incorporate both types of electron correlation, were conducted using the ORCA^43^ program. The chosen active space was the set of atomic-like f-orbitals in addition to the molecular orbitals resulting from combining actinide f orbitals and O_yl_ p orbitals. The active space involves 10 molecular orbitals, 2π_u_, 3σ_u_, 1ϕ_u_, 1δ_u_, 4σ_u_^*^, and 3π_u_^*^, as shown by Denning in Figure 1 of his study.^44^ The main atomic orbital composition of these molecular orbitals is given in Table S1. A similar selection was adopted by Gendron et al.^45^ for neptunyl(VI) complexes. This resulted in CASSCF(8,10) configurations for NpO_2_^+^ and CASSCF(9,10) configurations for PuO_2_^+^. A more complete active space would include non bonding, bonding and antibonding molecular orbitals resulting of the inclusion of 6d actinide orbitals and their combination with O_yl_ 2s and 2p orbitals. However, this would lead up to a 16-orbital active space what increased dramatically the number of configurations, e.g., for the PuO_2_^+^ case the number of configurations for the (9,10) one is ∼7 × 10^3^, whereas for the (15,16) is ∼4.9 × 10^6^. Bearing in mind that we have to deal with the hydrated actinyl aqua ions, both optimizing their geometries and producing a significant numbers of single points to build the interaction potentials, we have adopted the 10-orbital active space also used in precedent studies of complexes of similar sizes.^28,45^ Since the triplet and quartet ground states for NpO_2_^+^ and PuO_2_^+^, respectively, are degenerate, calculations were carried out using a state average over the degenerate states excluding excited states. The perturbational step of the calculation was done using quasi-degenerate perturbation theory. The basis sets used were ma-def2-TZVP for O, def2-SVP for H^46,47^ and SD(60,MWB)//def-TZVP for actinoids.^48^ The calculations were accelerated using the RI and RIJK pseudospectral methods with “autoaux” auxiliary basis sets. Due to the lack of analytical gradients, geometry optimizations were conducted numerically by evenly changing the M–O_yl_ and M–O_I_ distances in a 2D grid with a step of ∼0.005 Å. The structure was assumed to be optimized when the energy of the predicted optimized structure within the grid differs from the QM value obtained for such optimized geometry in less than 10^–5^*E*_h_, otherwise a reduced 2D grid, with a smaller step, around this point is computed to estimate the minimum.

### Interaction Potentials for AnO_2_^+^ in Water

To describe the interactions of PuO_2_^+^ and NpO_2_^+^ in aqueous solution, we have developed a procedure based on our statistical implementation of the hydrated ion concept,^49,50^ particularly adapted for the case of monovalent molecular cations.^36,51^Figure 1 displays a sketch of the interaction potentials involved in the system definition.

The basic idea is that the hydrated ion^53^ is the representative species interacting with the bulk water, ([AnO_2_(H_2_O)_*n*_]^+^–H_2_O) by means of a hydrated ion–bulk water potential (HIW). For this aim, the QM interaction energy, *E*_int_^QM^, is described by two potentials, of which one is the TIP4P potential^52^ that describes the interaction among the first-shell and bulk water molecules and the other collects the interactions between the bulk water molecules and the actinyl cation. The functional form is
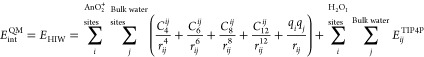
1

The interaction of the molecular cation with its first hydration shell (IW1) was parametrized by a series of *r*^–*n*^ terms with *n* = 4, 6, 8, and 12 plus the Coulombic term.
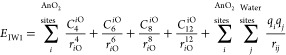
2

At this point it should be noted that the first-shell water molecules are ruled by an interaction potential with the actinyl cation (IW1) different from that of bulk water molecules (HIW). This supplies a refined definition of the interaction within the aqua ion and with the bulk water which allows classical optimized geometries of the aqua ion, as those shown in Table 1, very close to the QM ones, but requiring computational times of seconds instead of tens of hours. The shortcoming associated with this methodological advantage is the fact that no exchange of water molecules between the first-shell and the bulk must occur. Along the MD simulations no water molecule release from the aqua ions to the bulk was observed. Finally, to describe the intrinsic dynamics of the actinyl cation we have adopted for the intramolecular cation (IMC) potential a new functional form with respect to our previous development.^36−38,51^ We have replaced an interatomic potential based on a power series, as in the *E*_HIW_ or *E*_IW1_ functional forms, by an anharmonic potential to describe the An–O_yl_ bonds and a harmonic potential for the bending.
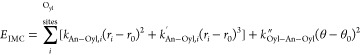
3

**Figure 2:**
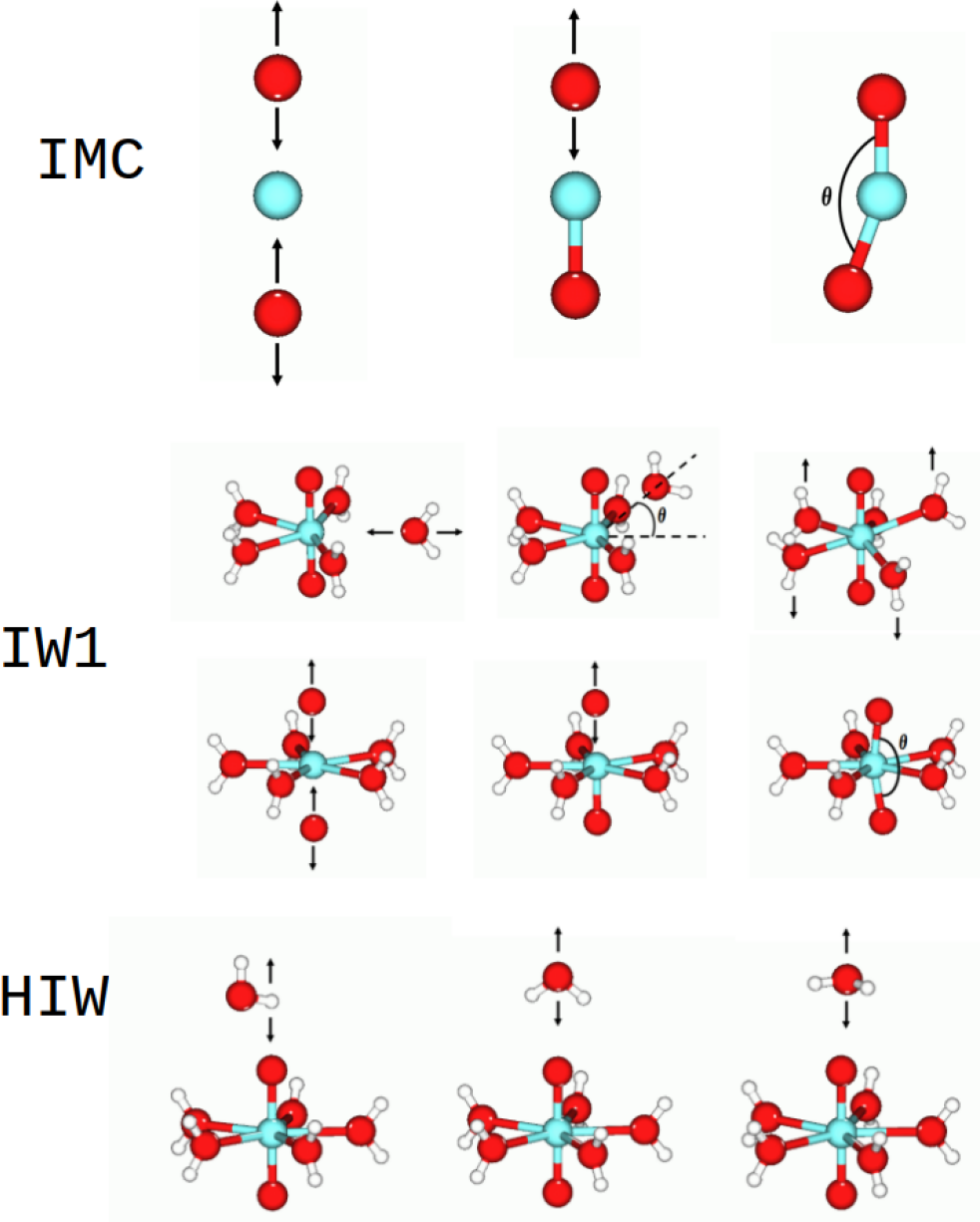
Representative type of structures employed to fit the potentials defined in the system: IMC (intramolecular cation interaction), IW1 (molecular cation–water first-shell interactions), and HIW (hydrated ion–bulk water interactions).

Figure 2 displays some representative structures used to build the intermolecular potentials. A total of 64 structures were used for the HIW potential, while 220 structures were used for the IW1 and 87 were used for the IMC. For this development, it has been assumed that the aqua ions are pentahydrates. Figures S1 and S2 show the fitting of the set of PuO_2_^+^ and NpO_2_^+^ potentials. Potential coefficients are given in Tables S2–S5, which include their corresponding standard deviations.

To check the fitted potentials, we have examined the interaction energy of a bulk water molecule approaching the hydrated actinyl from different regions. Figure S3 shows the good correlation between the QM interaction energy and the value predicted by the force field. It must be stressed that these structures were taken from 50 snapshots of an MD simulation where the closest water molecule to the actinyl aqua ion in each angular region was taken.

Given that for the PuO_2_^+^ aqueous solution, several authors^14,15,20^ have pointed out that its aqua ion is a tetrahydrate instead of a pentahydrate, a specific force field assuming that the hydrated ion is [PuO_2_(H_2_O)_4_]^+^ was also developed. A fitting procedure similar to that explained for the pentahydrated plutonyl was applied to define the new IMC potential, and the new PuO_2_^+^ polarization on the tetrahydrate was reflected in the Pu and O_yl_ effective charges collected in Table S2. Figure S4 shows the goodness of the fit by comparing the QM interaction energy with the values derived from the IW1 and HIW potentials of the force field.

### Molecular Dynamics Simulations

MD simulations were run in a similar way to our previous studies on actinyls.^36,38,51^ A single hydrated actinyl ion, [AnO_2_(H_2_O)_*n*_]^+^ (An = Np and Pu), and 1490 TIP4P water molecules were placed in a cubic box at the experimental water density. The simulations were run at 300 K in the *NVT* ensemble using the Noosé–Hoover thermostat with τ = 0.5 ps. Nonbonded interactions were cut at 12 Å, and the Ewald sum was used for the computation of electrostatic interactions. The equations of motion were integrated using a 0.5 fs time step for a total simulation time of 5 ns. All simulations were run using a modified version of DL_POLY Classic^54^ which includes the functional forms of the force field employed. The convergence of MD trajectories has been checked by the analysis of structural, energetic and dynamic properties of the actinyl cations as shown in refs (36), (38), and (51).

The translational self-diffusion coefficient of actinyls, *D*_AnO2_, has been obtained using mean-square displacements (MSD).^55^ This function was computed using multiple time origins up to half of the simulation period for each series. This procedure is particularly appropriate when describing the mobility of only one ion in the system. The 5 ns trajectory was employed to get an average value of *D*_AnO2_, analyzing five series of 1 ns. From it, the average value and an estimation of the error by its standard deviation were conducted. To compute ion hydration enthalpies, Δ*H*_hydr_, *NPT* MD simulations of 1 ns production at 300 K were also conducted. These simulations used the Nosé–Hoover thermostat and barostat with τ = 0.5 ps in both cases. The standard deviation of the average configurational enthalpies was computed by the blocking average method proposed by Flyvbjerg and Petersen^56^ for the error estimation on correlated data.

### Simulated XAS Spectra

A total of 500 evenly spaced configurations of [AnO_2_(H_2_O)_*n*_]^+^ were extracted from 1 ns MD trajectories, i.e., the time interval between two consecutive snapshots is 2 ps, which guarantees noncorrelated statistical information. It has been checked that the use of longer MD trajectories leads to the same simulated spectrum. The configurations included water molecules up to the first solvation shell since we have found the second shell to have no influence on the spectra. Average *L*_III_-edge spectra were obtained from the individual spectra using the FEFF code (version 9.6)^39^ including multiple scattering up to four-legged paths. Details of the spectrum simulation method can be found elsewhere.^23,31,38,51^ An example of the FEFF input files can be found in Figure S5. *S*_0_^2^ and Δ*E*_0_ values have been chosen in the simulated NpO_2_^+^ and PuO_2_^+^ spectrum in order to match the first resonance of the corresponding experimental spectrum.

### Experimental EXAFS Spectra

Experimental EXAFS spectra of NpO_2_^+^ and PuO_2_^+^ aqueous solutions have been recorded as described in ref (15). Revisited analysis of the previous published spectra was conducted with the ATHENA and ARTEMIS codes of Demeter 0.9.25 package^57^ in fluorescence mode for Np and in transmission mode for Pu.

## Results and Discussion

Table 1 collects the An–O_yl_ and An–O_I_ distances for the two hydrated actinyl cations, [AnO_2_(H_2_O)_*n*_]^+^ (*n* = 4 and 5), obtained at the QM level by the NEVPT2 method. Likewise, we have included the optimized geometry obtained by using the classical force fields developed (see “POTn (NEVPT2)” rows in the table). It must be underlined that the structural agreement between the QM and force field results is within the hundredth of angstrom. When average distances in solution (see “MD_POTn (300 K)” rows in the table) are considered, it is seen that the An–O_yl_ distance increases slightly ∼0.017 Å due to solvent effects. For PuO_2_^+^, the value of POT5(NEVPT2) is 1.805 Å, and that for MD_POT5(300 K) is 1.822 Å. For NpO_2_^+^, the corresponding values are 1.825 and 1.842 Å. When analyzing the solvent effects for the An–O_I_ distance the change is very small, on the order of 0.001 Å. Thus, Table 1 shows for the PuO_2_^+^ case that POT5(NEVPT2) gives 2.505 Å, and MD_POT5 (300 K) gives 2.507 Å. For the NpO_2_^+^ case, the corresponding values are 2.529 and 2.528 Å. As expected, for the hydrate in gas phase, the Np–O_yl_ distance is larger than that of the Pu–O_yl_ and the same trend is observed for the An–O_I_ bond. In water, the hydration effects do not change the gas-phase distance order. For the sake of comparison the scarce experimental data are also collected in Table 1. Our theoretical simulations agree fairly well with available experiments. In the plutonyl case, the experimental EXAFS data presented in this work are also in the narrow range of the previous data. As a matter of fact, no optimization geometry at the highly correlated NEVPT2 level of calculation had been previously reported for plutonyl, and as already observed for the neptunyl case in our previous work on actinyls,^38^ the most sensitive parameter to the electron correlation is the oxo bond. This effect involves a lengthening of the Pu–O_yl_ bond by 0.05 Å (1.81 Å this work and 1.76 Å with B3LYP),^58^ and a similar change is observed in this work for neptunyl (1.83 Å) and B3LYP (1.78–1.81 Å).^25,38,58^ This lengthening causes an An–O_I_ distance shortening of some hundreths of an angstrom. For the PuO_2_^+^ case, the value obtained is 2.51 Å (QM(NEVPT2) for CN = 5 row in Table 1) in this work, whereas the B3LYP values are 2.53 and 2.61 Å.^58,25^ For NpO_2_^+^, this work finds 2.52 Å (QM(NEVPT2) for CN = 5 row in Table 1), and previous B3LYP values are in the range of 2.55–2.61 Å.^25,38,58^ In the case of NpO_2_^+^, we can compare the performance of the new formulation of the IMC potential, based on harmonic and anharmonic functions, to describe the flexibility of the actinyl entity, with respect to the previous forms, based on a set of *r*^–*n*^ powers. The optimized geometry for [NpO_2_(H_2_O)_5_]^+^ using the new potential, POT5(NEVPT2), predicts distance changes smaller than 0.01 Å with respect to the previous values (see values in parentheses in the POT5(NEVPOT2) row).

Due to the sensitivity of the main geometrical parameters to the different QM methods, we have explored for the two actinyl aqua ions the An–O_*y*_ and An–O_I_ distance change when going from HF to NEVPT2(*n*,10) computations. Table 2 collects these two optimized distances obtained from different methods. When passing from the HF to CASSCF(n,10) wave functions, the inclusion of static electron correlation provides multideterminantal wave functions that are eigenfunctions of the total electron spin operators, a quartet for the plutonyl(V) aqua ion and a triplet for the neptunyl(V) aqua ion. This effect shortens by 0.02–0.03 Å the An–O_yl_ and lengthens the An–O_I_ by ∼0.1 Å. The inclusion of the dynamic correlation into these CASSCF wave functions by means of the NEVPT2 method leads to a significant increase of the oxo-bonds by ∼0.07 Å what contributes to a strong decreasing of the An–O_I_ by ∼0.12 Å. For the multideterminantal wave functions of [PuO_2_(H_2_O)_5_]^+^ and [NpO_2_(H_2_O)_5_]^+^ when passing from the CASSCF(*n*,10) to the NEVPT2(*n*,10), the first-order correction to the wave function is expanded over a set of properly chosen multireference functions which correctly take into consideration the two–electron interactions occurring among the active electrons.^42^ The MP2 method induces a slight increases of the An–O_yl_ bond and a strong decrease of the An–O_I_ with respect to the HF results. This unbalanced effects must be reflecting the fact of perturbing an uncorrelated unrestricted-spin wave function. We have included in the table the case of the uranyl pentahydrate, a closed-shell case, computed at the HF and MP2 level. In this case it is observed how for the one-determinantal wave function, the dynamic electron correlation introduced by MP2 leads to a lengthening of both distances.

**Table 2:** Optimized Distances (Å) of [NpO_2_(H_2_O)_5_]^+^, [PuO_2_(H_2_O)_5_]^+^, and [UO_2_(H_2_O)_5_]^2+^ Obtained via Different QM Methods

	[PuO_2_(H_2_O)_5_]^+^		[NpO_2_(H_2_O)_5_]^+^		[UO_2_(H_2_O)_5_]^2+^	
method	Pu–O_yl_	Pu–O_I_	Np–O_yl_	Np–O_I_	U–O_yl_	U–O_I_
HF	1.77	2.53	1.78	2.54	1.74	2.40
CASSCF(*n*,10)	1.74	2.63	1.76	2.64		
NEVPT2(*n*,10)	1.81	2.51	1.83	2.52		
MP2	1.78	2.42	1.80	2.48	1.78	2.45
B3LYP	1.78	2.55	1.79	2.55		

The B3LYP method includes an approach of the wave function dynamically correlated via the electron density estimation what leads to modest increases of the An–O_yl_ bond as well as of the An–O_I_. The fact we are dealing with multireferencial wave functions makes hard to separate the effects that static and dynamic electron correlation causes on the geometries. Nevertheless, it is generally accepted that the most rigorous way to undertake this type of systems is via a methodology which allows a balanced combination of static and dynamic electron correlation such as the NEVPT2 method provides.^40,41^

**Figure 3:**
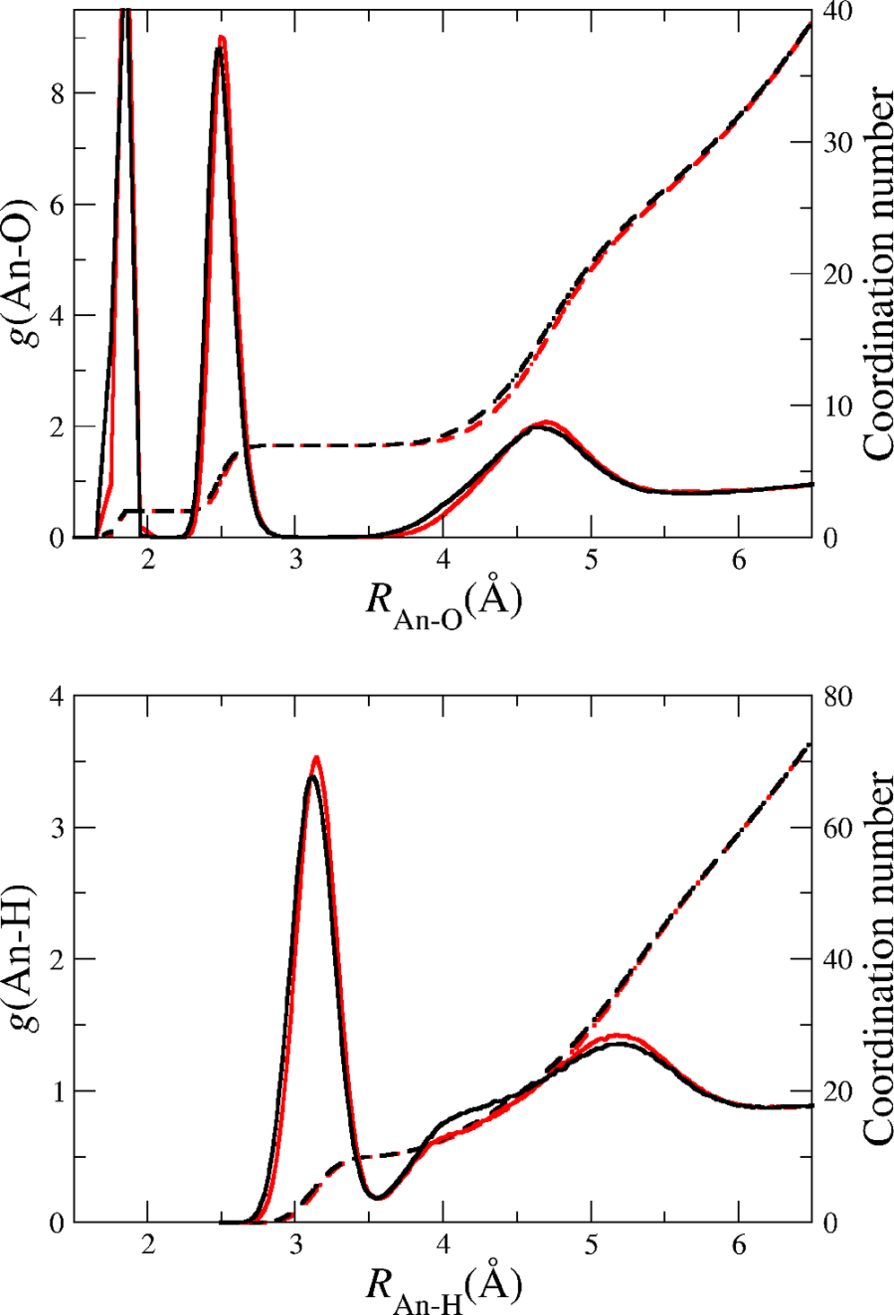
An–O (top) and An–H (bottom) radial distribution functions and their coordination numbers for Np(V) (red) and Pu(V) (black) in aqueous solution as obtained from the MD simulations.

Figure 3 shows the An–O and An-H RDFs for the NpO_2_^+^ (red lines) and PuO_2_^+^ (black lines) cations in aqueous solution derived from the MD simulations. The RDFs of both cations are quite similar, only a slight shifting toward longer distances in the mean values is observed (see Table 1 MD_POTn (300 K) rows) when passing from PuO_2_^+^ to NpO_2_^+^. This is a consequence of the native quantum-mechanical trend observed in the minimized pentahydrates. Thus, the QM gap of *R*_An–Oyl_ between NpO_2_^+^ and PuO_2_^+^ hydrates is 0.025 Å, whereas the RDFs shows a gap of 0.02 Å. The trend of the An–O_I_ parameter is similar to that of the An–O_yl_: the Np–O_I_ distance is ∼0.02 Å longer than that of Pu–O_I_, and the gap in solution is similar because the mean distance changes induced by solvation are only ∼0.001 Å. The number of water molecules in the second shell is ∼21 for the two cations, these values are similar to those corresponding to their divalent cations (see Figures 2 and 3 of ref (51)).

**Figure 4:**
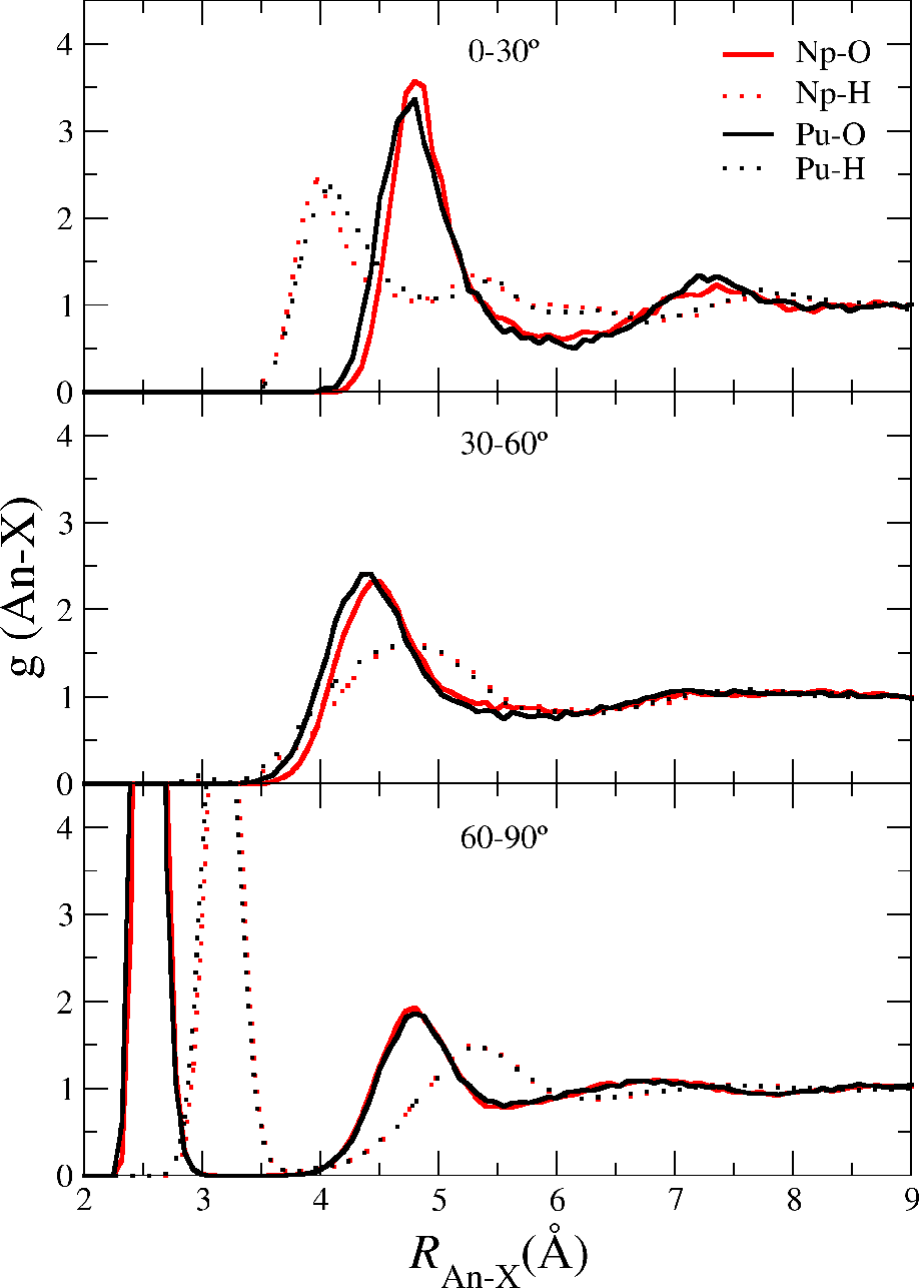
An–O and An–H angle-solved RDFs Np(V) (red) and Pu(V) (black) in aqueous solution as obtained from the MD simulations.

Figure 4 shows the distribution of water molecules around the different angular regions in one hemisphere that can be defined taking advantage of the system symmetry. The equatorial region (60–90° and 90–120°) presents two well-defined shells; the An–O and An–H first peaks correspond to the first hydration shell, already shown in Figure 3, as well as a second hydration shell that integrates to ∼9.2 molecules centered at 4.8 Å for the oxygen atoms. The comparison for each angular region of the An–O and An–H peak position sheds light on the relative orientation of water molecules. Thus, in the equatorial region, hydration shells take an ion-dipole orientation, because the An–H peak is shifted ∼0.7 Å from the An–O peak. In the intermediate zones (30–60° and 120–150°), the running integration number is ∼9 centered far from the actinyl, ∼4.5 Å, and the An–O and An–H peaks overlap, which means that the water molecules orientation is rather a compromise of their interactions among the molecular cation and the hydration water molecules. In the axial regions, ∼3.4 molecules are associated with the main peak which is centered at ∼4.5 Å from the actinide. This rather depopulated axial region shows that hydrogen atoms are closer to the metal cation than to the oxygen atoms due to the presence of O_yl_ atoms. Bearing in mind that the An–O_yl_ distance is ∼1.8 Å, the mean distance of one water hydrogen atom to the O_yl_ atom is ∼2.2 Å. This weak hydrogen bond pattern was not found in the previous cases studied of divalent actinyl.^51^ The last two regions can be envisaged as hydration structures that build the condensed medium around the aqua ion as well as they solvate slightly the actinyl cation.

Table 3 collects a set of energetic and dynamical properties of the monovalent aqueous solutions. The hydration enthalpies agree well with the estimated experimental values given by Gibson et al.^59^ It must be indicated that the sensitivity of PuO_2_^+^ electron wave function to its close environment have caused a large uncertainty in the hydration energy among different authors^29^ as indicated by Ryzhkov et al.^35^ in their recent study on Pu complexes in water. Taking into account the uncertainties, one can conclude that the hydration enthalpy of both cations is almost the same. Regarding the aqua ion dynamics, their size-corrected diffusion coefficient values for neptunyl and plutonyl are also quite similar. The calculated ion mobility is affected by the water mobility, which depends on the water model employed, in our case TIP4P. This model overestimates the water diffusion coefficient, 3.3 × 10^–5^ cm^2^ s^–1^,^60^ as the experimental value is 2.3 × 10^–5^ cm^2^ s^–1^. For this reason, a better test is needed to compare the values normalized by the water self-diffusion coefficient. The *D*_An_/*D*_w_ values are 0.44 and 0.43 for NpO_2_^+^ and PuO_2_^+^, respectively. Tiwari et al.^61^ have computed the diffusion coefficient for the monovalent actinyls using the SPC/E water model, their normalized values of the size-corrected *D*_An_/*D*_w_ are 0.46 and 0.45 for NpO_2_^+^ and PuO_2_^+^, respectively. Their corresponding values for the divalent actinyls, NpO_2_^2+^ and PuO_2_^2+^, computed in our previous work^38^ with the same methodology but using a B3LYP-based force field are 0.38, that represents a low limit of diffusion for PuO_2_^+^ and NpO_2_^+^ as they are singly charged. Simonin et al.^62^ have determined experimentally for UO_2_^2+^ at infinite dilute aqueous solution a normalized value of 0.30. Our theoretical normalized value for the divalent uranyl, which was also computed in our previous work^38^ was 0.37.

**Table 3:** Energetic and Dynamical Properties Calculated from the MD Simulations

property	NpO_2_^+^	PuO_2_^+^
Δ*H*_hyd_ (kcal mol^–1^)	–166 ± 3	–165 ± 3
*ΔH*_hyd_^exp^(59) (kcal mol^–1^)	–180 ± 20	–178 ± 20
*D*_An_ (10^–5^ cm^2^ s^–1^)	1.1 ± 0.2	1.1 ± 0.2
*D*_An_^corr^ (10^–5^ cm^2^ s^–1^)	1.4 ± 0.2	1.4 ± 0.2
*D*_An_/*D*_W_	0.44 ± 0.04	0.43 ± 0.04

Another illustrative test of the actinyl dynamics in aqueous solutions is the analysis of the most representative vibrational normal modes. Table 4 shows the symmetric (1A_1_) and asymmetric (A_2_) An–O_yl_ stretching normal modes, the O_yl_–An–O_yl_ bending (E_1_) and the water breathing stretching (2A_1_). To account for the solvent effects, the gas-phase frequencies of the two pentahydrates have been included in the table. Interestingly, one can observe that for the two actinyl stretching modes the solvation induces redshifts of their frequencies of about 15–20 cm^–1^. This is a consequence of the interactions with second-shell water molecules in the intermediate and axial regions. On the contrary, solvent effects induce a blueshift of ∼50 cm^–1^ in the water-breathing mode. This is due to the strong aqua ion–water interactions in the equatorial region, where second-shell water molecules causes a compactness of the first-shell water molecules, then increasing the corresponding frequency of the water breathing vibrational mode. Only three experimental frequencies have been reported in the literature;^63−65^ the error of our estimation is smaller than 4% and the sequence predicted by our potentials is the same than the experimental one.

**Table 4:** Experimental and MD Normal Mode Frequencies (cm^–1^)

frequencies	method	*E*_1_	2*A*_1_	1*A*_1_	*A*_2_
[NpO_2_(H_2_O)_5_]^+^	MD (gas phase)	393	251	819	870
	MD (solution)	227	305	798	853
	exp^63,64^			767	824
[PuO_2_(H_2_O)_5_]^+^	MD (gas phase)	441	250	780	828
	MD (solution)	276	296	764	811
	exp^65^			748	

**Figure 5:**
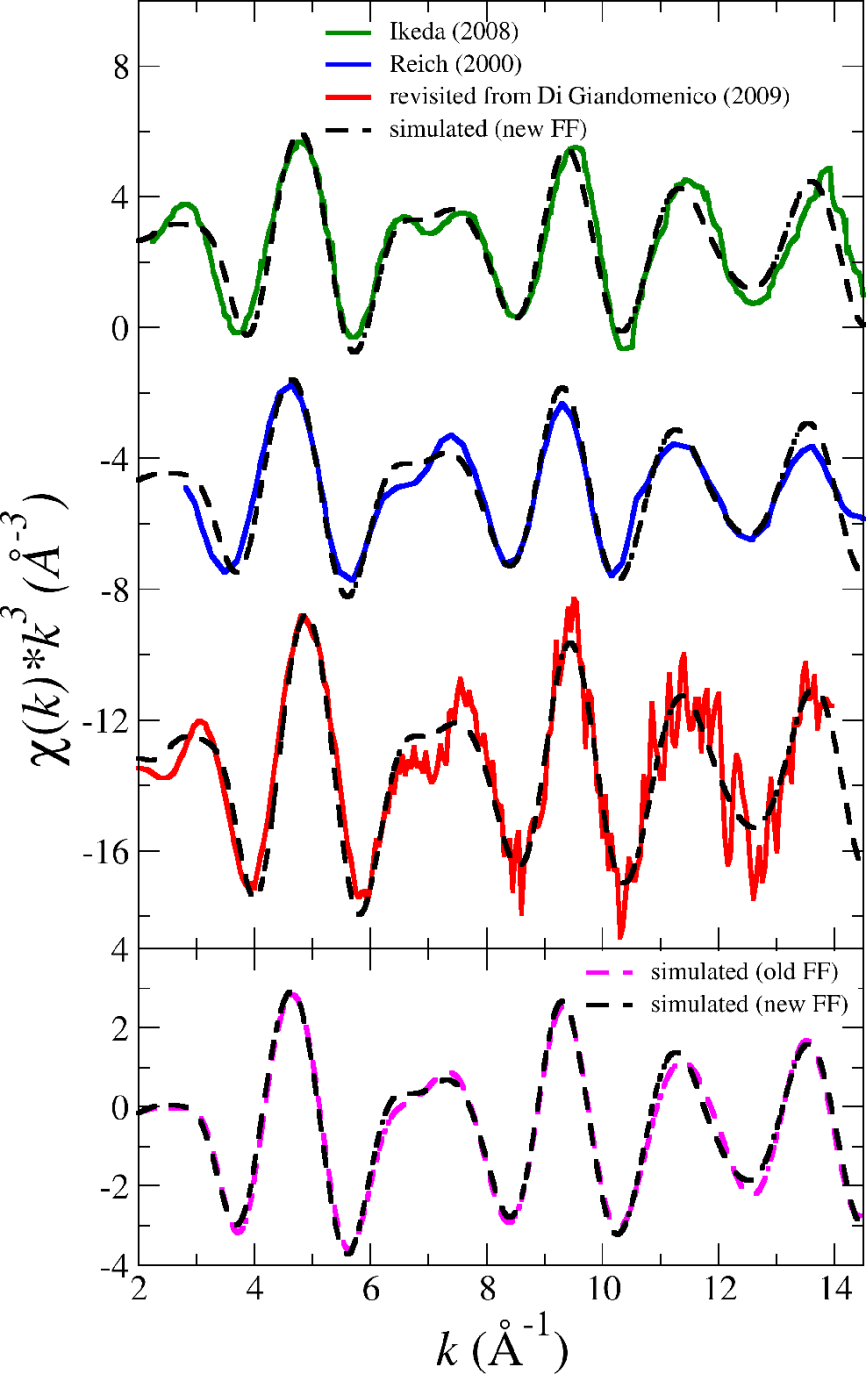
Top: Simulated (dashed black line) vs experimental (solid line green (ref (12)), blue (ref (9)), red (reanalyzed from ref (15))) Np L_III_-edge *k*^3^-weighted EXAFS spectra for NpO_2_^+^ in water. Bottom: Comparison between the two simulated EXAFS spectra computed by means of the new NEVPT2 force field developed in this work (black) and the force field developed in a previous work (magenta).^38^

**Figure 6:**
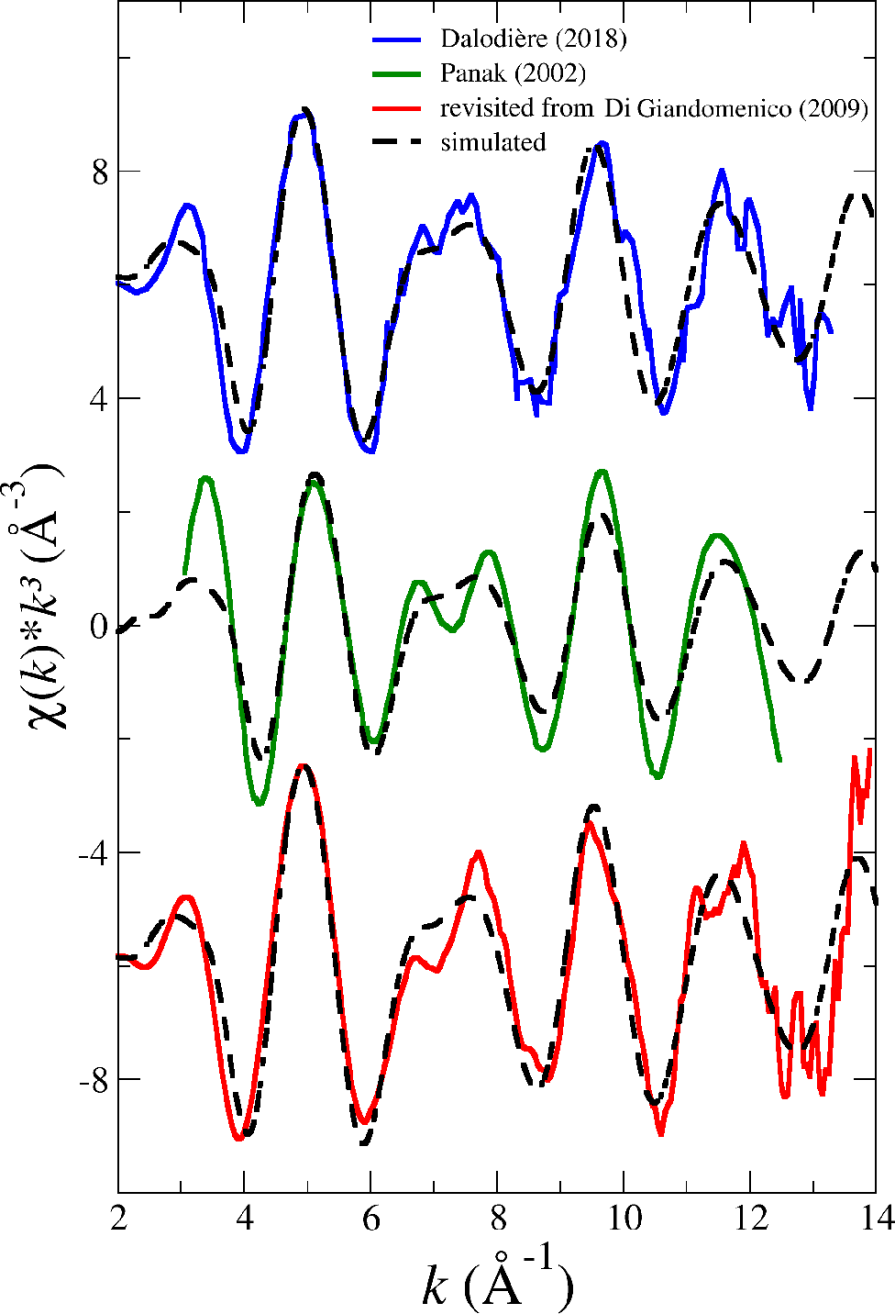
Simulated (dashed black line) vs experimental (solid line blue (ref (20)), green (ref (14)) and red (reanalyzed from ref (15))) Pu L_III_-edge *k*^3^-weighted EXAFS spectra for PuO_2_^+^ in water.

Figures 5 and 6 display the comparison of the experimental EXAFS spectrum reported in the literature from several authors for NpO_2_^+^ and PuO_2_^+^ in dilute aqueous solutions, together with the revisited spectra previously reported by Di Giandomenico et al.^15^ In the NpO_2_^+^ case, we can see the reasonable agreement of our simulated spectrum with the three experimental ones.^9,12,15^ Because the experimental difficulties for the X-ray absorption spectrum recording, the difference among the experimental spectra is similar to that observed for the experimental–theoretical comparison. In the bottom of Figure 5 we have included the simulated EXAFS spectrum obtained using our previous NEVPT2-based intermolecular potential.^38^ This spectrum almost matches the spectrum obtained in this work which employs a simplified version of the intramolecular actinyl potential (IMC).

Regarding the PuO_2_^+^ EXAFS spectra, Figure 6 also shows that the differences among them are similar to the relative discrepancy of our simulated spectrum with the three experimental spectra. From the set of EXAFS measurements carried out by one of us on the actinyls in a previous work,^15^ the revisited PuO_2_^+^ spectrum recorded in transmission mode has been analyzed and included in Figure 6. The spectrum is similar to the spectrum published in that article, although signal/noise is higher as well as global intensity. (cf., “revisited spectrum” in Figure 6 with Pu(V)/HClO_4_ in Figure 4 of ref (15)). The complicated shape of the EXAFS spectrum is reproduced well by the simulated one in the five oscillations experimentally recorded.

The striking question is the fact that the *S*_0_^2^ values needed to match the main oscillations (maximum at *k* = 5 Å^–1^) are small, 0.7 for the transmission mode spectrum,^15^ 0.6 for that of Dalodière et al.,^20^ and 0.45 for that of Panak et al. one.^14^ In the case of the NpO_2_^+^, the values needed are in the range of 0.7–0.9. This fact might be related to the reduction of the coordination number from 5 water molecules in NpO_2_^+^ aqueous solution to 4 in the PuO_2_^+^ case.

To find out on this issue we have undertaken three additional analysis: (i) quantum-mechanical computation of the relative stability of the two hydrates in water at the same level of calculation employed to develop the force field, (ii) experimental fitting of the previously recorded plutonyl EXAFS spectrum assuming the constraint of a hydration number 4 or 5, and (iii) development a force field for PuO_2_^+^ in water based on a tetrahydrate cation and the analysis of results derived from the corresponding MD simulation at 300 K.

A direct procedure to estimate quantum-mechanically the relative stability of [PuO_2_(H_2_O)_4_]^+^ and [PuO_2_(H_2_O)_5_]^+^ in water is the computation of the equilibrium
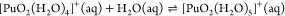
4

This equation can be envisaged as the difference between the PuO_2_^+^ hydration free energy corresponding to the formation of the tetrahydrate and pentahydrate aqua ions in water
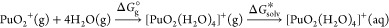
5
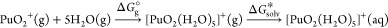
6
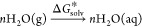
7

The hydration free energy of PuO_2_^+^ is then computed by the addition of the gas-phase hydrate formation, Δ*G*_g_^°^, its solvation in water, Δ*G*_solv_^*^, computed by means of the continuum polarizable model,^66^ CPCM,^67^ as implemented in the ORCA program,^43^ the vaporization free energy of *n* water and the standard state correction associated with the gas phase-solution transfer, as given by Goddard et al.^68^ The values of the hydration energy are −124 and −128 kcal/mol for the tetra- and pentahydrate, respectively. Table S6 collects the different contributions to these estimations. From these data, the free energy of eq 4 is −4 kcal/mol, which indicates the preference for the pentahydration of PuO_2_^+^ in water from a quantum-mechanical semicontinuum model of solvation.^69^

**Figure 7:**
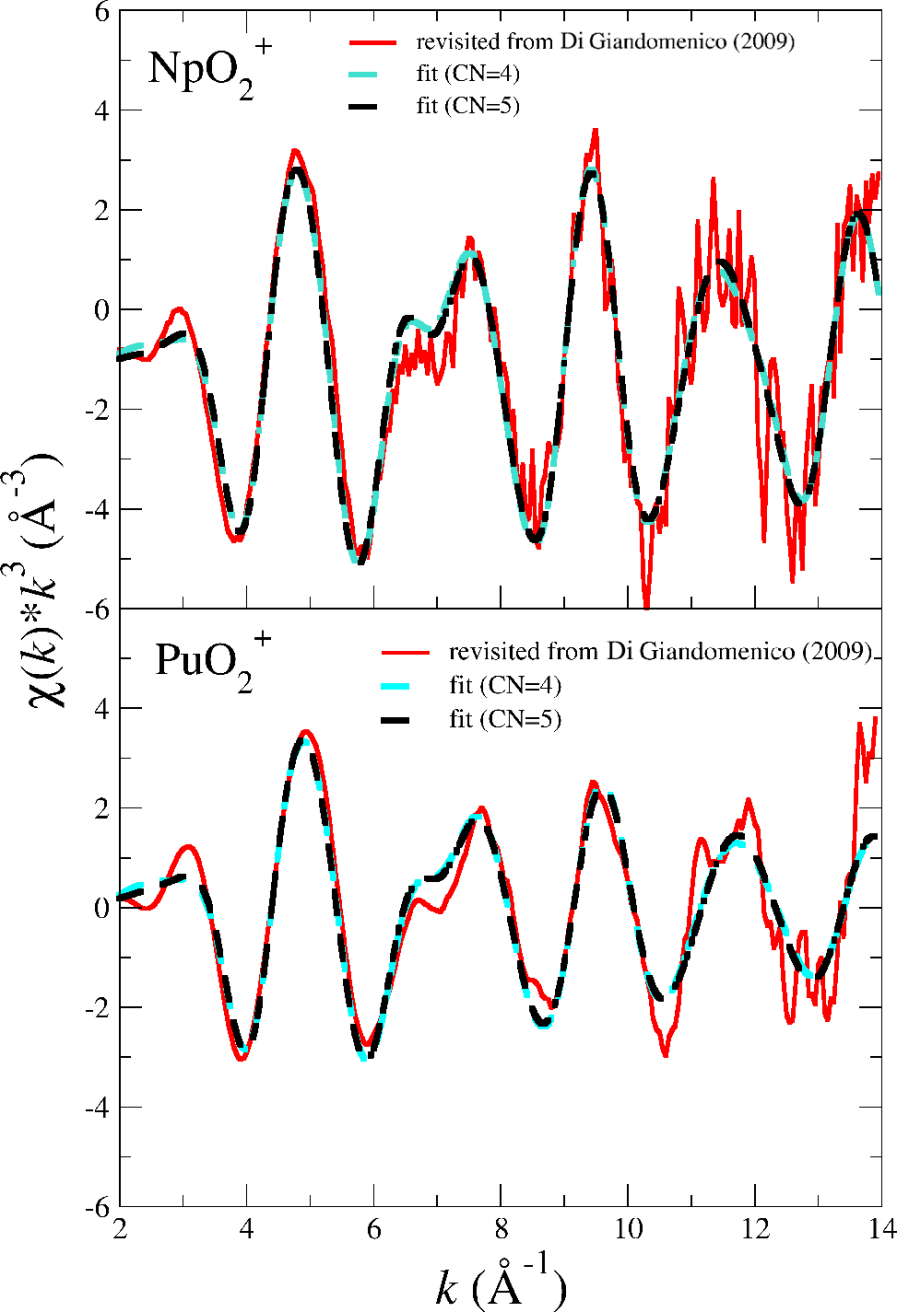
Np (top) and Pu (bottom) L_III_-edge *k*^3^-weighted EXAFS spectra for NpO_2_^+^ and PuO_2_^+^ in water: experimental (red solid line)^15^ and fits assuming a hydration number of four (cyan dashed line) or five (black dashed line).

Figure 7 shows the revisited experimental *L*_3_-edge *k*^3^-weighted EXAFS spectra of NpO_2_^+^ and PuO_2_^+^ ^15^ and their fits obtained when the first hydration shell is constrained to 4 (cyan dashed line) or 5 (black dashed line). It is observed that the two fits are very similar, and that conclusion is verified by examining Table 5, which collects the main parameters of the fits. This leads to the fact that the assumption of a given coordination number does not change the values of the fitted parameters, since the quality of the fits are almost the same. As already indicated, the sensitivity of the EXAFS for these spectra with such a low signal/noise ratio, due to the high experimental complexity of the measurements, blurs the subtle discrimination of one unit coordination number. Regarding the main An–O distances, one can compare the sequence of distances derived from our MD simulations (MD_POT5 (300 K) rows in Table 1) with the EXAFS fit of Table 5. The trend with the change of actinoid is the same, i.e., *R*(NpO_yl_) > *R*(PuO_yl_) and *R*(NpO_I_) > *R*(PuO_I_). Even more, the distance changes when going from Np to Pu given by MD simulations and the EXAFS fittings are close: Δ*R*_AnOyl_ is −0.02 Å (MD) and −0.03 Å (EXAFS fit); Δ*R*_AnOI_ is −0.02 Å (MD) and −0.04 Å (EXAFS fit).

**Table 5:** EXAFS Best-Fit Parameters of NpO_2_^+^ and PuO_2_^+^ in HClO_4_

fit parameters	NpO_2_^+^ (CN = 4)	NpO_2_^+^ (CN = 5)	PuO_2_^+^ (CN = 4)	PuO_2_^+^ (CN = 5)
*R*(An–O_yl_) (Å)	1.83(1)	1.83(1)	1.80(1)	1.80(1)
σ^2^(An–O_yl_) (Å^2^)	0.0003	0.0000	0.0019	0.0015
*R*(An–O_I_) (Å)	2.51(1)	2.51(1)	2.47(1)	2.47(1)
σ^2^(An–O_I_) (Å^2^)	0.0034	0.0044	0.0061	0.0074
*S*_0_^2^	0.9	0.8	0.8	0.7
*E*_0_ (eV)	6.0	5.6	6.4	6.1
*R*_factor_ (%)	2.5	2.6	4.9	6.5

**Figure 8:**
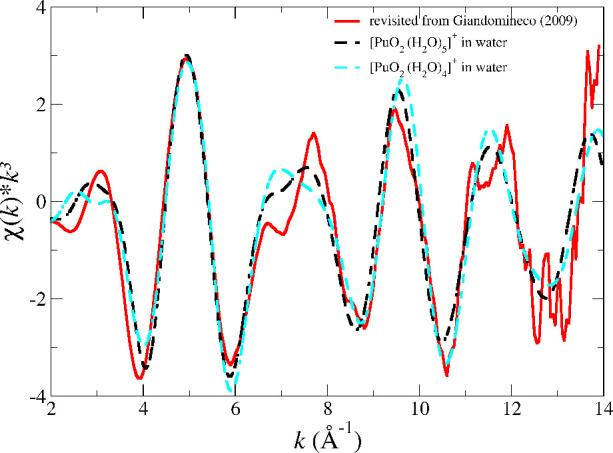
Simulated Pu L_III_-edge *k*^3^-weighted EXAFS spectra for PuO_2_^+^ in water using the restricted 4 first-shell water molecules force field, POT4(NEVPT2) (cyan), or the pentahydrate force field, (POT5(NEVPT2) (black) vs. the experimental one.^15^

The final analysis deals with the use of a force field developed on the basis of the intramolecular and intermolecular interactions of [PuO_2_(H_2_O)_4_]^+^. When running a free MD simulation at 300 K, the plutonyl cation hydration always evolves to a pentahydrate. Pomogaev et al.^58^ in their MD simulations of a set of monovalent actinyl cations showed how the initial tetrahydrate cation, defined as starting hydrated ion, became a pentahydrate when the system evolved. To get a deeper insight into this issue, we envisage a strategy to get a set of snapshots derived from a simulation of a PuO_2_^+^ aqueous solution, where the cation first-shell was formed by only four water molecules. Toward this aim, we added to our HIW force field for the plutonyl tetrahydrate an additional repulsive Pu–O term, (*C*/*r*^–8^), that prevented the presence of bulk water molecules inside the first shell. This penalty function vanishes beyond the first shell such as the first-shell–second-shell interactions are those provided by the original force field built from the tetrahydrate. Table 1 collects the main geometrical parameters of the optimized clusters, POT4(NEVPT2) for CN = 4, that compare fairly well with the corresponding QM optimizations of the same hydrates (*R*(PuO_yl_) is 1.800 Å (QM) and 1.795 (POT4) Å; *R*(PuO_I_) is 2.448 Å (QM) and 2.450 Å (POT4)). The Pu–O and Pu–H RDFs for the MD simulation using POT4(NEVPT2) are plotted in Figure S6, as well as the RDFs derived of the POT5(NEVPT2). The mean values derived from these POT4(NEVPT2) RDFs are also collected in Table 1 and show the same slight changes due to bulk solvent effects already observed for the POT5(NEVPT2) RDFs. Figure 8 compares the simulated EXAFS spectrum of PuO_2_^+^ in water obtained by the force field developed under the assumption of a pentahydration, which was already compared with the experimental spectra in Figure 6 and the spectrum obtained with the force field built imposing a tetrahydration. For the sake of comparison, the experimental PuO_2_^+^ EXAFS spectrum revisited from Di Giandomenico et al.^15^ has also been included in Figure 8. Two important facts can be drawn from this figure. The first one is that the tetrahydrate-derived EXAFS does not reproduce the spectrum shape in the peculiar 6–8 Å^–1^ region. The second one is that the intensity of both spectra is similar, although the coordination number, 5 versus 4, of their first hydration shell could lead to expect a higher intensity for the first spectrum. Certainly, the similarity between both spectra shows the great difficulty to discriminate a hydration number when experimental signal/noise ratio is low.

The relationship between the change of shape of the PuO_2_^+^ EXAFS spectrum in the 6–8 Å^–1^ region and the Pu–O_yl_ and Pu–O_I_ distances deserves a final comment. Hydration effects on the aqua ions described by means of the MD simulations, induce changes on the distances which are in the order of 0.01–0.02 Å. The corresponding changes when going from [PuO_2_(H_2_O)_4_]^+^ to [PuO_2_(H_2_O)_5_]^+^ are much more important, in particular, for the An–O_I_ distance that increases by ∼0.05 Å. This is already observed in the QM(NEVPT2) and in the intermolecular potentials developed POT5/4(NEVPT2) optimized structures. Since solvent effects are small, the gap of the Pu–O_I_ distance between the tetra- and the pentahydrate holds up in the in-solution simulations MD-POT4/5 (300 K) giving rise to the different shape of the EXAFS spectrum in the 6–8 Å^–1^ region. This subtle change is responsible for the good agreement found and supports the prevalence of the PuO_2_^+^ pentahydration in water. Interestingly, it can be seen how the experimental fittings shown in Table 5 give the same An–O_I_ distance for both coordination numbers in order to provide a good reproduction of such challenging region.

## Concluding Remarks

The use of highly correlated wave functions to build the force fields of the paramagnetic actinyls, NpO_2_^+^ and PuO_2_^+^, in aqueous solutions has been shown to improve the accuracy of physicochemical properties of the systems. In particular, the sensitivity of EXAFS spectrum shape to the structural features points out that the comparison of experimental and simulated spectra is a valuable tool to validate the microscopical structure provided by the statistical simulations.

The structural differences between these actinyl cations in aqueous solutions are small. The quantum mechanical description of the aqua ions are already small: *R*(Np–O_yl_) = 1.83 Å versus *R*(Pu–O_yl_) = 1.81 Å, and *R*(Np–O_I_) = 2.52 Å and *R*(Pu–O_I_) = 2.51 Å. Given that these actinyl aqua ions are monovalent, the impact of the rest of the solvent on their geometries is small, changing only slightly the internal parameters of the aqua ions. They are pentahydrates. This conclusion is based on the quantum-mechanical and statistical descriptions of PuO_2_^+^, the similarity observed at the same calculation level for NpO_2_^+^, where the hydration number is widely accepted as 5 and the good agreement with the experimental EXAFS spectra. The tetracoordination proposal based on EXAFS fitting could be biassed by the general low intensity of the recorded spectrum signal, for instance, when compared to the neptunyl case. To the generally accepted uncertainty of ±1 units in the coordination number provided by the EXAFS fitting, we must add the intrinsic experimental difficulties joined to the preparation and recording of these hazardous complexes radioactive samples. Modelization of ionic solutions with ad-hoc intramolecular potentials has helped to refine the analysis of intriguing radioactive species in water and validate future uses of these potentials to further simulations of these radioactive species in water. In particular, the revisiting of previously studied lower computational level actinyl divalent cations,^37,38^ such as PuO_2_^2+^, NpO_2_^2+^, and AmO_2_^2+^, as well as actinoid aqua ions,^70^ appear as challenging next steps in the study of the actinides in aqueous solutions.

## Supporting Information Available

Description of the intermolecular potential formalism. Tables with parameters force field obtained for AnO_2_^+^–H_2_O (M = Np, Pu). Figures showing the representative set of structure types employed in the fitted potentials. Plots showing the correlation between the QM interaction energies and the predicted values by the developed force field. Examples of input FEFF files used for the XAS spectrum simulations. Details of the QM computation of the PuO_2_^+^ tetrahydrate and pentahydrate equilibrium. Pu–O and Pu–H RDFs of the MD simulation conducted with the POT4(NEVPT2) potential (PDF)
