# Supplementary material for: A Coupled EXAFS–Molecular Dynamics Study on PuO2+ and NpO2+ Hydration: The Importance of Electron Correlation in Force-Field Building
Source: Inorg Chem. 2022 May 26;61(23):8703–14. doi: 10.1021/acs.inorgchem.2c00461 (PMC9199009; doi:10.1021/acs.inorgchem.2c00461)
Supplement: Supplementary file 1 — ic2c00461_si_001.pdf [file ic2c00461_si_001.pdf]

SUPPORTING INFORMATION

A coupled EXAFS–Molecular Dynamics study on the $PuO_2{}^+$ and $NpO_2{}^+$ hydration: the importance of the electron correlation in the force field building

Gema Raposo-Hernández[a], José M. Martínez[a], Rafael R. Pappalardo[a], Christophe Den Auwer[b] and Enrique Sánchez Marcos[a]

(a) *Departamento de Química Física, Universidad de Sevilla, 41012 Sevilla, Spain.*

e-mail:sanchez@us.es

(b) *Université Côte d'Azur, CNRS, ICN, 06108 Nice, France*

Table S1: Atomic Orbital composition (in %) of the active space MOs.

CAS MO	AO (Pu)		AO (O_{yl})	
	p	f	p	d
$2\pi_u$	2.9	23.0	36.7	0.1
$3\sigma_u$	2.1	49.9	23.1	0.0
$1\phi_u$	0.0	99.8	0.0	0.0
$1\delta_u$	0.0	99.0	0.0	0.0
$4\sigma_u^*$	7.2	53.3	16.9	1.3
$3\pi_u^*$	0.5	80.5	8.6	0.7

Table S2: Effective charges of $[AnO_2{\cdot}(H_2O)_5]^+(aq)$. Water molecules have the same structure and partial charges as the TIP4P model. The electron density used to fit the charges of An and Oyl is that of CASSCF.

Partial Charge (a.u.)			
	$NpO_2(H_2O)_5^+]$	$[PuO_2(H_2O)_5^+]$	$[PuO_2(H_2O)_4^+]$
q_{An}	2.82	2.75	2.593
$q_{O_{yl}}$	-0.91	-0.875	-0.7965
q_O	0.0	0.0	0.0
q_H	0.52	0.52	0.52
q_q	1.04	1.04	1.04

Table S3: Coefficients of the IW1 interaction potentials (POT5(NEVPT2)). Their units are kcal mol^{-1} Å^{n} and for root mean square kcal mol^{-1}.

Coefficients	$NpO_2(H_2O)_5^+]$	$[PuO_2(H_2O)_5^+]$		$NpO_2(H_2O)_5^+]$	$[PuO_2(H_2O)_5^+]$
$C_4^{\mathrm{AnO_I}}$	5.55673648	5.55673648	$C_4^{\mathrm{AnH_I}}$	0.0000	0.0000
$C_6^{\mathrm{AnO_I}}$	-15352.7184	-9024.89041	$C_6^{\mathrm{AnH_I}}$	887.824562	887.824562
$C_8^{\mathrm{AnO_I}}$	100288.826	61833.0743	$C_8^{\mathrm{AnH_I}}$	-13541.1870	-13541.1870
$C_{12}^{\mathrm{AnO_I}}$	-499414.791	-259807.920	$C_{12}^{\mathrm{AnH_I}}$	484910.935	484910.935
$C_4^{\mathrm{O_{yl}O_I}}$	-0.0000	-0.0000	$C_4^{\mathrm{O_{yl}H_I}}$	0.0	0.0
$C_6^{\mathrm{O_{yl}O_I}}$	7170.36089	1847.29987	$C_6^{\mathrm{O_{yl}H_I}}$	0.0	0.0
$C_8^{\mathrm{O_{yl}O_I}}$	-30587.4687	-75.7692528	$C_8^{\mathrm{O_{yl}H_I}}$	0.0	0.0
$C_{12}^{\mathrm{O_{yl}O_I}}$	169473.952	8357.73271	$C_{12}^{\mathrm{O_{yl}H_I}}$	0.0	0.0
RMSE	1.008	0.6724			

Table S4: Coefficients of IMC intermolecular potential (POT5(NEVPT2).

	$NpO_2(H_2O)_5^+]$	$[PuO_2(H_2O)_5^+]$
$k_{\text{An-Oyl}}$ (kcal mol^{-1} Å^{-2})	530.0589	481.0200
$k'_{\text{An-Oyl}}$ (kcal mol^{-1} Å^{-3})	-557.0657	-524.1588
$k_{\text{Oyl-An-Oyl}}$ (kcal mol^{-1} degrees^{-2})	21.1089	37.0448
r_0 (Å)	1.773345	1.757742
θ_0 (degrees)	180.0	180.0

Table S5: Coefficients of the HIW interaction potentials (POT5(NEVPT2)). Their units are kcal mol^{-1} Å^{n} and for root mean square kcal mol^{-1}

Coefficients	$NpO_2(H_2O)_5^+]$	$[PuO_2(H_2O)_5^+]$		$NpO_2(H_2O)_5^+]$	$[PuO_2(H_2O)_5^+]$
$C_4^{\mathrm{AnO_W}}$	-0.0000	0.000	$C_4^{\mathrm{AnH_W}}$	0.0000	0.0000
$C_6^{\mathrm{AnO_W}}$	3351.5862	3351.5862	$C_6^{\mathrm{AnH_W}}$	0.0000	0.0000
$C_8^{\mathrm{AnO_W}}$	0.0000	0.0000	$C_8^{\mathrm{AnH_W}}$	0.0000	0.0000
$C_{12}^{\mathrm{AnO_W}}$	0.0000	0.0000	$C_{12}^{\mathrm{AnH_W}}$	0.0000	0.0000
$C_4^{\mathrm{O_{yl}O_W}}$	-3.53294671	-3.53294671	$C_4^{\mathrm{O_{yl}H_W}}$	0.0	0.0
$C_6^{\mathrm{O_{yl}O_W}}$	-3590.73553	-2682.00915	$C_6^{\mathrm{O_{yl}H_W}}$	0.0	0.0
$C_8^{\mathrm{O_{yl}O_W}}$	23714.9539	12860.3146	$C_8^{\mathrm{O_{yl}H_W}}$	1159.64124	1475.42600
$C_{12}^{\mathrm{O_{yl}O_W}}$	0.00000000	0.00000000	$C_{12}^{\mathrm{O_{yl}H_W}}$	0.0	0.0
RMSE	1.825	0.250			

Computation of reaction free energy of the hydration of the Plutonyl(V) tetrahydrate to Plutonyl(V) pentahydrate.

$$[\mathrm{PuO_2(H_2O)_4}]^+\,(\mathrm{aq}) + \mathrm{H_2O}\,(\mathrm{aq}) \xrightarrow{\Delta \mathrm{G}^*(\mathrm{hydr})} [\mathrm{PuO_2(H_2O)_5}]^+\,(\mathrm{aq}) \tag{1}$$

can be computed as the difference between the solvation free energy of the plutonyl(V) tetrahydrate

$$\mathrm{PuO_2}^+\,(\mathrm{g}) + 4\,\mathrm{H_2O}\,(\mathrm{aq}) \xrightarrow{\Delta \mathrm{G}^*_{\mathrm{sol}}} [\mathrm{PuO_2(H_2O)_4}]^+\,(\mathrm{aq}) \tag{2}$$

and that of the plutonyl(V) pentahydrate

$$\mathrm{PuO_2}^+\,(\mathrm{g}) + 5\,\mathrm{H_2O}\,(\mathrm{aq}) \xrightarrow{\Delta \mathrm{G}^*_{\mathrm{sol}}} [\mathrm{PuO_2(H_2O)_5}]^+\,(\mathrm{aq}) \tag{3}$$

Then,

$$\Delta G^*(hydr) = \Delta G^*_{sol}([\mathrm{PuO_2(H_2O)_5}]^+\,) - \Delta G^*_{solv}([\mathrm{PuO_2(H_2O)_4}]^+\,) - \Delta G^*_{solv}(\mathrm{H_2O}\,) \tag{4}$$

These amounts can be computed by means of the following steps:

$$n\,\mathrm{H_2O}\,(\mathrm{aq}) \xrightarrow{-\Delta \mathrm{G}^*_{\mathrm{solv}}} n\,\mathrm{H_2O}\,(\mathrm{g}) \tag{5}$$

$$\mathrm{PuO_2}^+\,(\mathrm{g}) + \mathrm{nH_2O}\,(\mathrm{g}) \xrightarrow{\Delta \mathrm{G}^0_g} [\mathrm{PuO_2(H_2O)}_n]^+\,(\mathrm{g}) \tag{6}$$

$$[\mathrm{PuO_2(H_2O)}_n]^+\,(\mathrm{g}) \xrightarrow{\Delta \mathrm{G}^*_{\mathrm{solv}}} [\mathrm{PuO_2(H_2O)}_n]^+\,(\mathrm{aq}) \tag{7}$$

Then the solvation free energy of a plutonyl(V) cluster of n water molecules can be expressed by

$$\Delta G^*_{sol}([\mathrm{PuO_2(H_2O)}_n]^+\,(aq)) = \Delta G^*_{solv}([\mathrm{PuO_2(H_2O)}_n]^+\,(g)) + \Delta G^0_g(\mathrm{PuO_2}^+\,(g)) - n\Delta G^*_{solv}(\mathrm{H_2O}\,(g)) \tag{8}$$

Table S6: Contributions to the solvation free energy (kcal mol^{-1}) of PuO_2^+ tetrahydrate and pentahydrate.

Free energy	n=4	n=5
ΔG_g^0 (PuO_2^+ (g))	-75.2	-79.9
ΔG_{solv}^* ($[PuO_2(H_2O)_n]^+$ (g))	-51.3	-51.4
$\Delta G_{state-corr}^*$ ($[PuO_2(H_2O)_n]^+$)	-17.1	-21.4
-n ΔG_{solv}^* (H_2O (g))	19.6	24.5
ΔG_{sol}^* ($[PuO_2(H_2O)_n]^+$ (aq))	-124.0	-128.2

Table S7: Coefficients of the IW1 interaction potentials for the PuO_2^+ tetrahydrate (POT4(NEVPT2)). Their units are $kcal\,mol^{-1}\,Å^{n}$ and for root mean square kcal mol^{-1}

Coefficients	$[PuO_2(H_2O)_4^+]$		$[PuO_2(H_2O)_4^+]$
$C_4^{AnO_I}$	5.55673650	$C_4^{AnH_I}$	0.0000
$C_6^{AnO_I}$	-11903.0507E+05	$C_6^{AnH_I}$	887.824562
$C_8^{AnO_I}$	77557.7357E+05	$C_8^{AnH_I}$	-13541.1870
$C_{12}^{AnO_I}$	-332481.513E+06	$C_{12}^{AnH_I}$	484910.935
$C_4^{O_{yl}O_I}$	-0.0000	$C_4^{O_{yl}H_I}$	0.0
$C_6^{O_{yl}O_I}$	1847.29987	$C_6^{O_{yl}H_I}$	0.0
$C_8^{O_{yl}O_I}$	-75.7692528	$C_8^{O_{yl}H_I}$	0.0
$C_{12}^{O_{yl}O_I}$	8357.73271	$C_{12}^{O_{yl}H_I}$	0.0
RMSE	1.10		

Table S8: Coefficients of the HIW interaction potentials for the tetrahydrate PuO_2^+ (POT4(NEVPT2)). Their units are kcal mol^{-1} $Å^n$ and for root mean square kcal mol^{-1}

Coefficients	$[PuO_2(H_2O)_4^+]$		$[PuO_2(H_2O)_4^+]$
$C_4^{AnO_W}$	0.000	$C_4^{AnH_W}$	0.0000
$C_6^{AnO_W}$	3351.5862	$C_6^{AnH_W}$	0.0000
$C_8^{AnO_W}$	66337.4175	$C_8^{AnH_W}$	0.0000
$C_{12}^{AnO_W}$	0.0000	$C_{12}^{AnH_W}$	0.0000
$C_4^{O_{yl}O_W}$	-3.53294671	$C_4^{O_{yl}H_W}$	0.0
$C_6^{O_{yl}O_W}$	-2682.00915	$C_6^{O_{yl}H_W}$	0.0
$C_8^{O_{yl}O_W}$	12860.3147	$C_8^{O_{yl}H_W}$	1475.42600
$C_{12}^{O_{yl}O_W}$	0.00000000	$C_{12}^{O_{yl}H_W}$	0.0
RMSE	1.20		

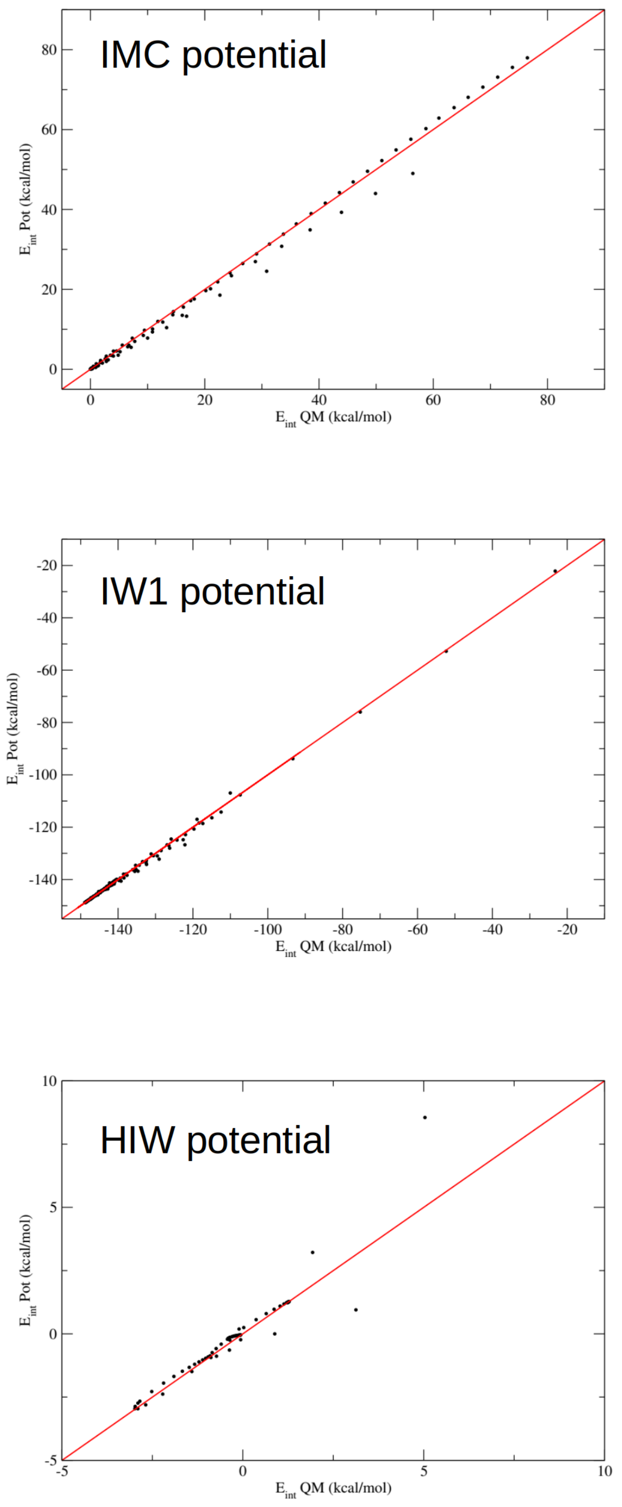

Figure S1: PuO_2^+–H_2O potential fitting. IMC, intramolecular cation potential; IW1, ion-water first shell potential; HIW, hydrated ion-water of the bulk potential.

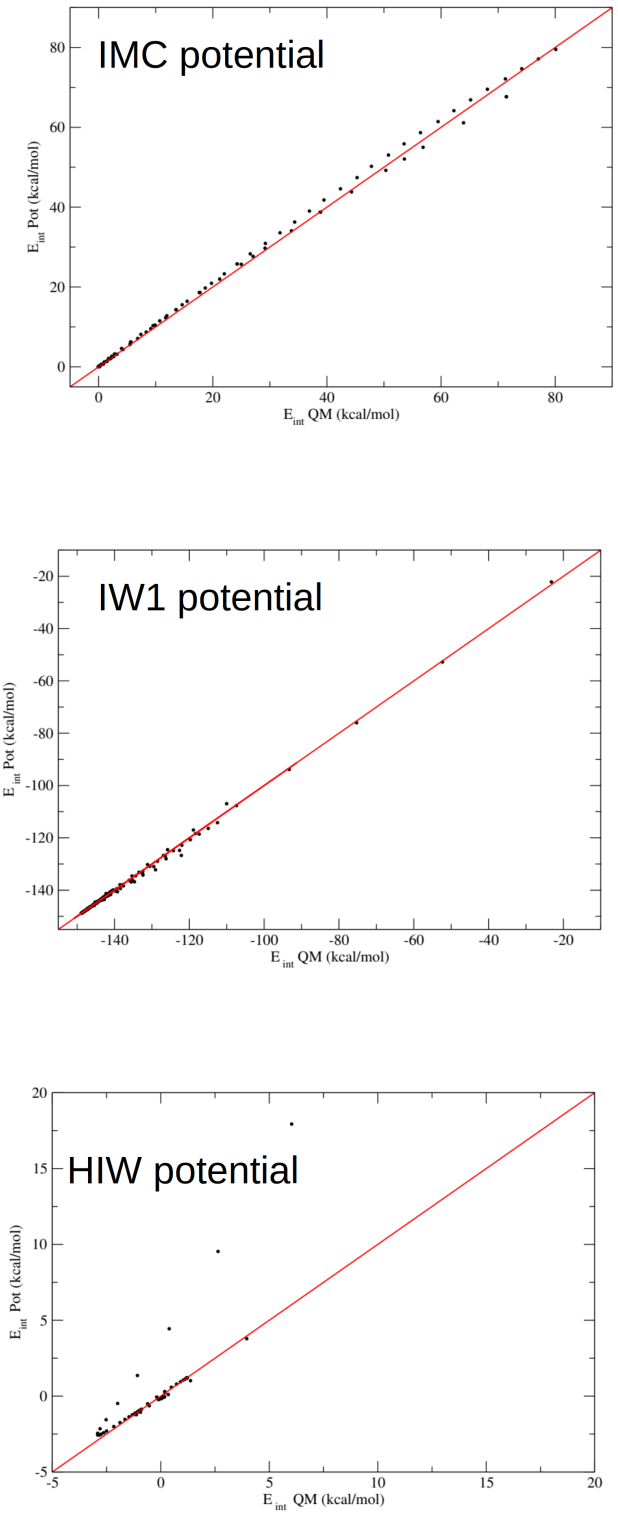

Figure S2: NpO_2^+–H_2O potential fitting. IMC, intramolecular cation potential; IW1, ion-water first shell potential; HIW, hydrated ion-water of the bulk potential.

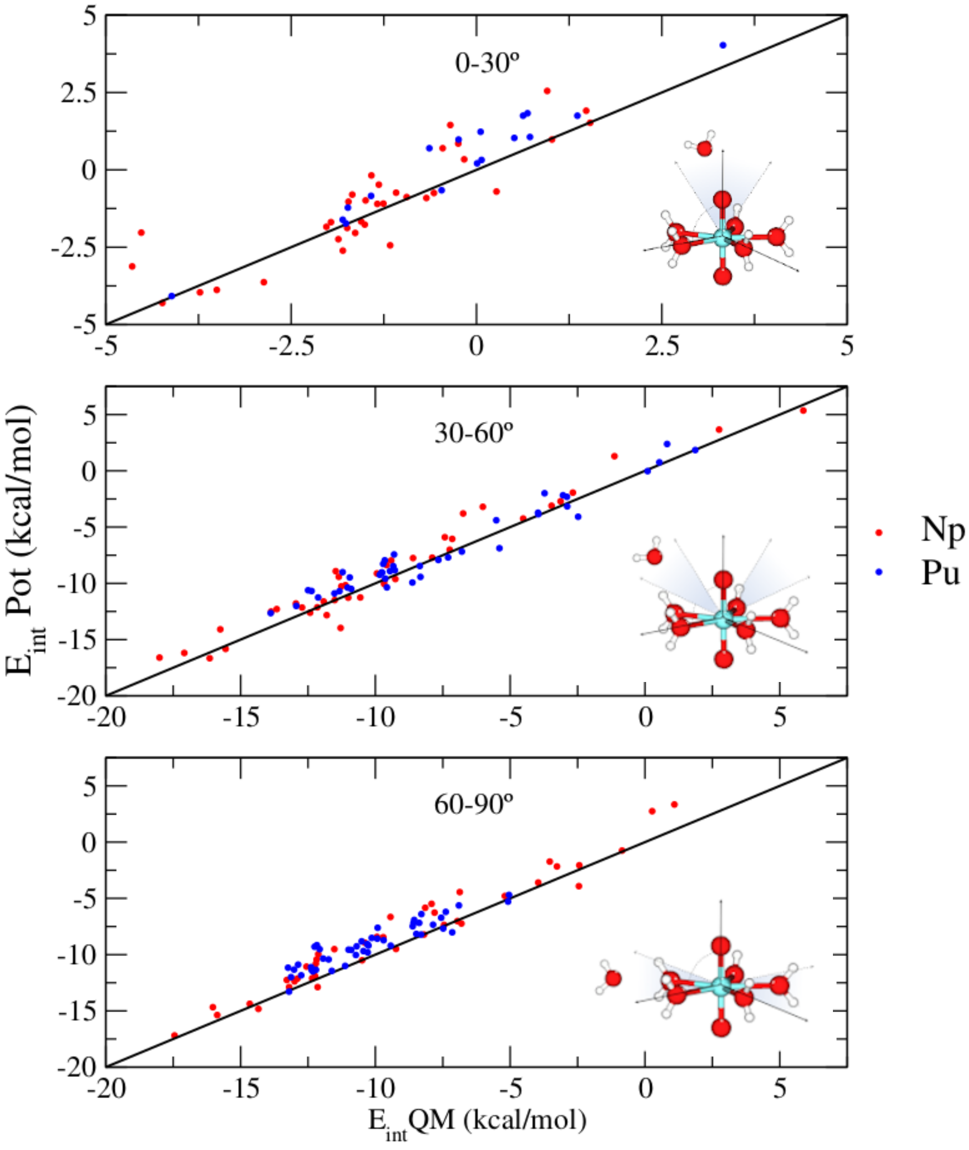

Figure S3: Potential vs. quantum mechanical interaction energy (kcal mol^{-1}) of a water molecule with the neptunyl (red points) or the plutonyl (blue points) aquaions in different regions. Standard deviations (kcal mol^{-1}) of the structures considered are: 0.8 (Np), 0.5 (Pu) (region 0-30°), 1.1 (Np), 1.0 (Pu) (region 30-60°) and 1.0 (Np), 0.8 (Pu) (region 60-90°)

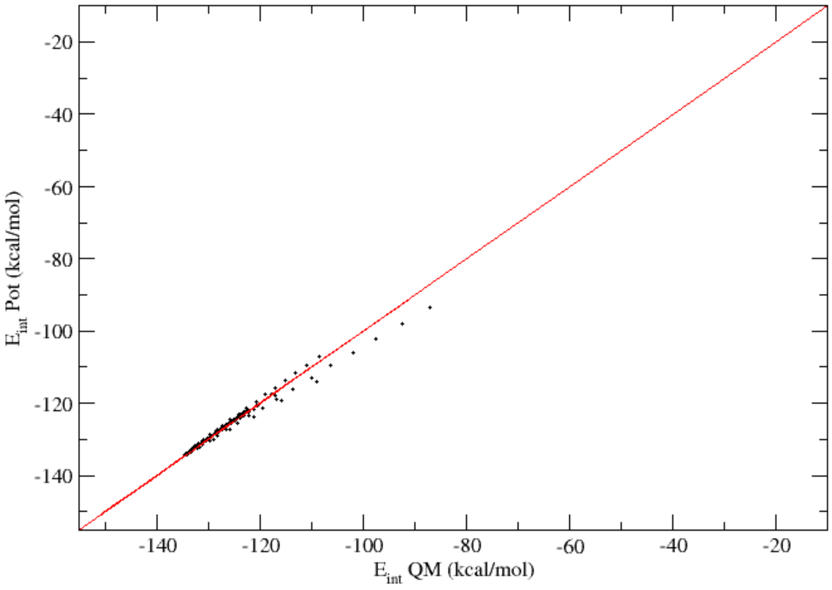

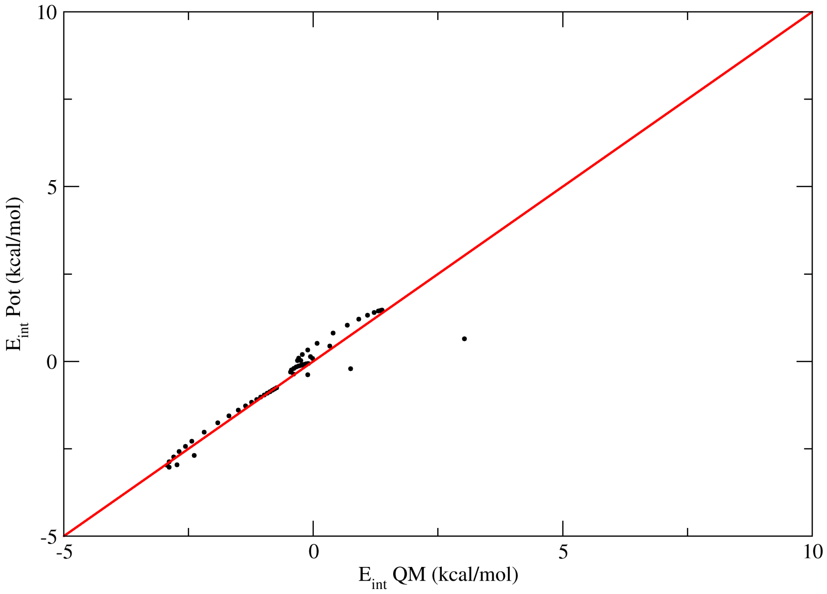

Figure S4: PuO_2^+–H_2O potential fitting assuming a tetracoordination (POT4(NEVPT2)) IW1 (top) and HIW (bottom) ion-water first shell potential

```
TITLE PuO2 5H2O

EDGE L3

 POTENTIALS
 *   ipot   z  label  l
       0    94  Pu    3   3
       1    8   Oyl   3   3
       2    8   O     3   3
       3    1   H     2   2

*         mphase,  mpath,  mfeff,  mchi
CONTROL  1   1   1   1   1   1
PRINT      0       0       0       1

EXCHANGE 0  0.0 0
SCF 6.0

COREHOLE RPA
TDLDA  1
CRITERIA 4.0  2.5

RPATH   6.0  ! ONLY for EXAFS
NLEG    4

S02 1.0
ATOMS
   0.0000000    0.0000000    0.0000000  0   Pu     0.0000000  0
   1.2810000    0.7488000   -1.1040000  1  Oyl     1.8494536  0
  -1.1400000   -0.8025000    1.1690000  1  Oyl     1.8193865  0
  -1.4360000    0.1742000   -1.9350000  2    O     2.4159194  0
  -1.3350000    0.3214000   -2.8760000  3    H     3.1869890  0
  -2.3080000   -0.2096000   -1.8440000  3    H     2.9616097  0
   0.6290000    1.4468000    1.8270000  2    O     2.4138766  0
   1.4170000    1.8685000    2.1710000  3    H     3.1956881  0
  -0.0290000    1.5755000    2.5092000  3    H     2.9629590  0
   0.5250000   -2.2522000   -0.9110000  2    O     2.4855484  0
   1.4640000   -2.3650000   -1.0630000  3    H     2.9776652  0
   0.1740000   -3.1427000   -0.9050000  3    H     3.2750365  0
  -1.5300000    2.4292000    0.0410000  2    O     2.8711659  0
  -2.4860000    2.4025000    0.0810000  3    H     3.4581445  0
  -1.3310000    3.2519000   -0.4060000  3    H     3.5371246  0
   2.2800000   -0.8584000    1.0350000  2    O     2.6469748  0
   3.0640000   -0.4286000    0.6920000  3    H     3.1702773  0
   2.5630000   -1.2477000    1.8630000  3    H     3.4053624  0
  -3.7160000   -1.1949000   -0.7200000  2    O     3.9692370  0
  -3.9830000   -2.1026000   -0.5760000  3    H     4.5405938  0
  -4.5020000   -0.6809000   -0.5380000  3    H     4.5848744  0
END
```

Figure S5: FEFF input file for the case of the plutonyl in water: EXAFS signal computation.

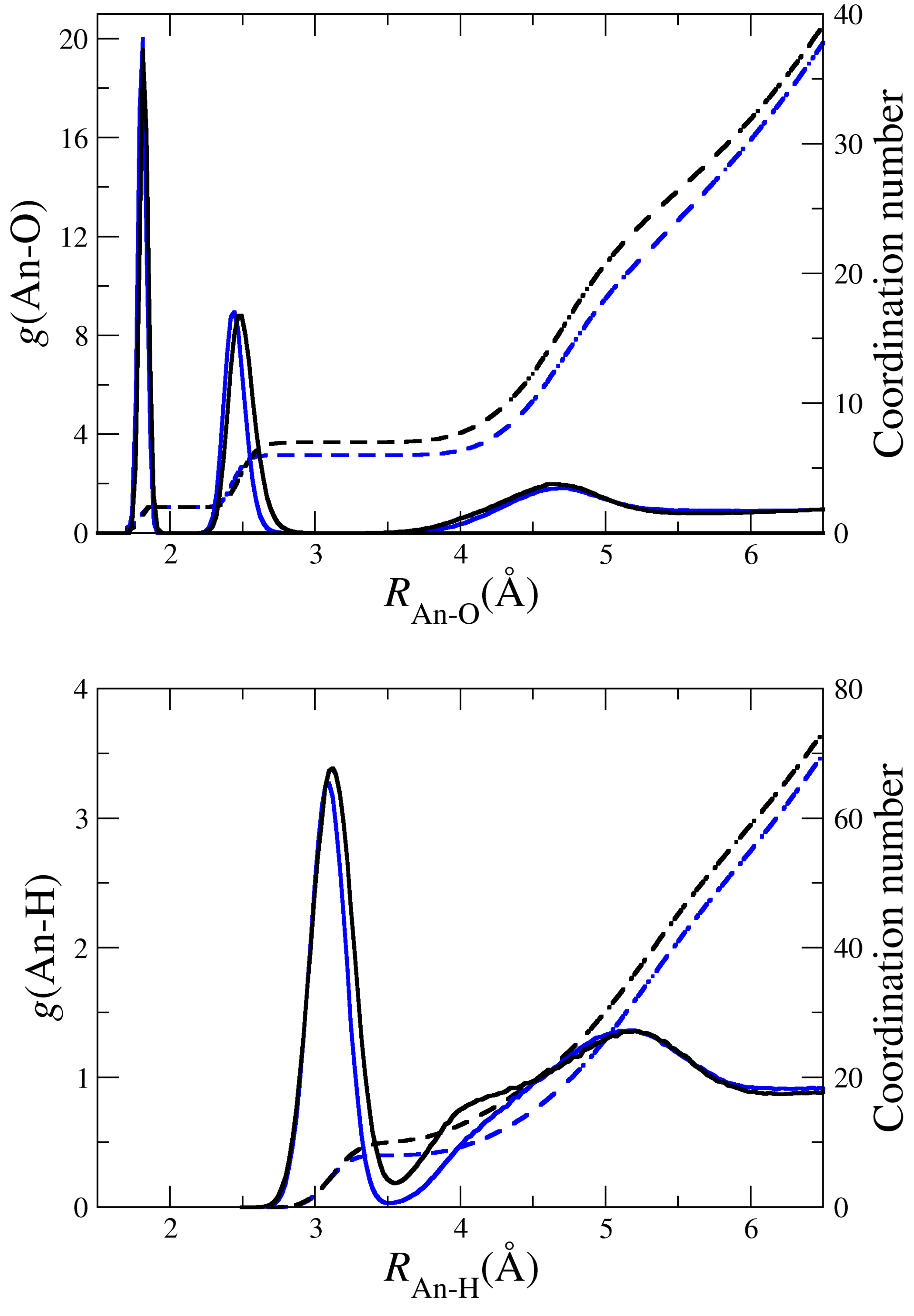

Figure S6: An-O and An-H RDFs and their coordination number of PuO_2^+ in water obtained by force field where the hydrated ion considered was a tetrahydrate (POT4(NEVPT2)) (blue) or a pentahydrate (POT5(NEVPT2) (black).
